# MCC950 Ameliorates Diabetic Muscle Atrophy in Mice by Inhibition of Pyroptosis and Its Synergistic Effect with Aerobic Exercise

**DOI:** 10.3390/molecules29030712

**Published:** 2024-02-04

**Authors:** Xiaoyu Yan, Pengyu Fu, Yimin Zhang, Dongmei Ling, Lewis Reynolds, Weicheng Hua, Zhiyuan Wang, Fangyuan Ma, Boxuan Li, Jingjing Yu, Yujia Liu, Lijing Gong, Enming Zhang

**Affiliations:** 1 Key Laboratory of Exercise and Physical Fitness of Ministry of Education, Beijing Sport University, Beijing 100084, China; yanxiaoyu57@163.com (X.Y.); yujingjing@bsu.edu.cn (J.Y.); 2 School of Sport Science, Beijing Sport University, Beijing 100084, China; fupy@nwpu.edu.cn (P.F.); 15279705047@163.com (D.L.); 2019210254@bsu.edu.cn (W.H.); zhiyuanw2024@163.com (Z.W.); 18563830595@163.com (F.M.); boxuanli@link.cuhk.edu.hk (B.L.); liuyujia89@163.com (Y.L.); 3 Department of Physical Education, Northwestern Polytechnical University, Xi’an 710072, China; 4 Department of Clinical Sciences in Malmö, Lund University Diabetes Centre, Lund University, 21428 Malmö, Swedenenming.zhang@med.lu.se (E.Z.); 5 NanoLund Center for NanoScience, Lund University, 22100 Lund, Sweden; 6 School of Life Sciences, Nankai University, Tianjin 300071, China; 7 School of Life Sciences, The Chinese University of Hong Kong, Hong Kong SAR 999077, China; 8 Institute of Physical Education, Jiangsu Normal University, Xuzhou 221116, China

**Keywords:** diabetes, muscle atrophy, MCC950, NLRP3, pyroptosis, aerobic exercise

## Abstract

Diabetic muscle atrophy is an inflammation-related complication of type-2 diabetes mellitus (T2DM). Even though regular exercise prevents further deterioration of atrophic status, there is no effective mediator available for treatment and the underlying cellular mechanisms are less explored. In this study, we investigated the therapeutic potential of MCC950, a specific, small-molecule inhibitor of NLRP3, to treat pyroptosis and diabetic muscle atrophy in mice. Furthermore, we used MCC950 to intervene in the protective effects of aerobic exercise against muscle atrophy in diabetic mice. Blood and gastrocnemius muscle (GAS) samples were collected after 12 weeks of intervention and the atrophic state was assessed. We initially corroborated a diabetic muscle atrophy phenotype in *db*/*db* mice (D) by comparison with control m/m mice (W) by examining parameters such as fasting blood glucose (D vs. W: 24.47 ± 0.45 mmol L^−1^ vs. 4.26 ± 0.6 mmol L^−1^, *p* < 0.05), grip strength (D vs. W: 166.87 ± 15.19 g vs. 191.76 ± 14.13 g, *p* < 0.05), exercise time (D vs. W: 1082.38 ± 104.67 s vs. 1716 ± 168.55 s, *p* < 0.05) and exercise speed to exhaustion (D vs. W: 24.25 ± 2.12 m min^−1^ vs. 34.75 ± 2.66 m min^−1^, *p* < 0.05), GAS wet weight (D vs. W: 0.07 ± 0.01 g vs. 0.13 ± 0.01 g, *p* < 0.05), the ratio of GAS wet weight to body weight (D vs. W: 0.18 ± 0.01% vs. 0.54 ± 0.02%, *p* < 0.05), and muscle fiber cross-sectional area (FCSA) (D vs. W: 1875 ± 368.19 µm^2^ vs. 2747.83 ± 406.44 µm^2^, *p* < 0.05). We found that both MCC950 (10 mg kg^−1^) treatment and exercise improved the atrophic parameters that had deteriorated in the *db*/*db* mice, inhibited serum inflammatory markers and significantly attenuated pyroptosis in atrophic GAS. In addition, a combined MCC950 treatment with exercise (DEI) exhibited a further improvement in glucose uptake capacity and muscle performance. This combined treatment also improved the FCSA of GAS muscle indicated by Laminin immunofluorescence compared to the group with the inhibitor treatment alone (DI) (DEI vs. DI: 2597 ± 310.97 vs. 1974.67 ± 326.15 µm^2^, *p* < 0.05) or exercise only (DE) (DEI vs. DE: 2597 ± 310.97 vs. 2006.33 ± 263.468 µm^2^, *p* < 0.05). Intriguingly, the combination of MCC950 treatment and exercise significantly reduced NLRP3-mediated inflammatory factors such as cleaved-Caspase-1, GSDMD-N and prevented apoptosis and pyroptosis in atrophic GAS. These findings for the first time demonstrate that targeting NLRP3-mediated pyroptosis with MCC950 improves diabetic muscle homeostasis and muscle function. We also report that inhibiting pyroptosis by MCC950 can enhance the beneficial effects of aerobic exercise on diabetic muscle atrophy. Since T2DM and muscle atrophy are age-related diseases, the young mice used in the current study do not seem to fully reflect the characteristics of diabetic muscle atrophy. Considering the fragile nature of *db*/*db* mice and for the complete implementation of the exercise intervention, we used relatively young *db*/*db* mice and the atrophic state in the mice was thoroughly confirmed. Taken together, the current study comprehensively investigated the therapeutic effect of NLRP3-mediated pyroptosis inhibited by MCC950 on diabetic muscle mass, strength and exercise performance, as well as the synergistic effects of MCC950 and exercise intervention, therefore providing a novel strategy for the treatment of the disease.

## 1. Introduction

Type-2 diabetes mellitus (T2DM) manifests as chronic hyperglycemia, impaired microcirculation and the dysfunction of various organs and tissues, for instance, muscle atrophy [1]. The onset of muscle wasting seriously affects the quality of life in diabetic individuals, while maintaining muscle homeostasis preserves overall body integrity and function [2]. However, the molecular mechanisms in and treatment strategies for diabetic muscular atrophy are less explored [3]. One of the prevailing ways to promote muscle hypertrophy and alleviate muscle atrophy is aerobic exercise, which enhances the protein metabolism balance within skeletal muscles [4], as well as reduces the levels of key inflammatory markers, such as Interleukin-6 (IL-6), C-reactive protein (CRP) and tumor necrosis factor-α (TNF-α) in T2DM [5]. Moreover, AMPK and PGC1α signaling pathways in muscles activated by such exercise facilitate adaptation to the inflammatory effects and metabolizing status, eventually resulting in a beneficial impact on mitigating muscle loss [6]. Yet, the anti-inflammatory pathways induced by exercise exhibit a high selectivity and diversity within the muscles; for instance, 8-week endurance training in T2DM patients raised the low levels of anti-inflammatory inhibitory nuclear factor kappa-B (IKB) in muscle [7] but remained unchanged in 16-week resistance training [8]. Therefore, the exploration of the unveiled mediators that are associated with the exercise-induced anti-inflammatory pathways in the muscle of T2DM is a potential strategy for the treatment of atrophy.

Nucleotide-binding and oligomerization domain (NOD)-like receptor family pyrin domain containing 3 (NLRP3) is the well-established inflammasome that switches various inflammatory responses. NLRP3 plays an important role in metabolic inflammatory diseases and participates in the progression of T2DM and its complications [9]. NLRP3 knockout mice exhibit higher glucose tolerance and insulin sensitivity, which prevents diet-induced diabetes [10]. NLRP3-mediated pyroptosis occurs in diabetic myocardial and glomerular endothelial cells, promoting the release of pro-inflammatory factors [9]. Moreover, the inhibition of NLRP3 inflammasome activation reduces inflammation-related atrophy and increases muscle strength [11]. While NLRP3 has been recognized as a vital regulator of skeletal muscle metabolism and implicated in the pathogenesis of muscle atrophy, its specific role in diabetic muscle atrophy remains unclear. Therefore, inhibition of the NLRP3 inflammasome may represent a new trend of anti-atrophy in inflammation-associated skeletal muscle [11], and identification of pharmacological NLRP3 inhibitors may be a beneficial strategy for the prevention and treatment of diabetic muscular atrophy.

MCC950 (C_20_H_24_N_2_O_5_S), a small molecule, shows a specific inhibitory effect on the NLRP3 by blocking classical and non-classical NLRP3 activation [12]. The protective effect of MCC950 has been verified in some diabetic animal models, such as diabetic nephropathy [13], diabetes-associated atherosclerosis [14], diabetic encephalopathy [15] and diabetic retinopathy [16]. In addition, MCC950 treatment mitigates impaired inflammation-induced muscle growth [17] and plays a beneficial role in Duchenne muscular dystrophy (DMD) [18]. The administration of MCC950 improves the muscle function, increases the fiber size and decreases muscle pro-inflammatory cytokines and pyroptosis in DMD [18]. However, the protective potential and underlying mechanisms in MCC950 against diabetic muscle atrophy have not been well investigated yet [19,20].

In diabetic adipose tissue, aerobic exercise alleviates insulin resistance and improves insulin sensitivity by inhibiting the expression of NLRP3 and IL-1β [21]. Javaid et al. [21] attempted to hypothesize that inhibiting NLRP3 could serve as a complementary treatment alongside exercise to ameliorate diabetic muscle atrophy. In this study, we implemented the injection of MCC950 to block NLRP3-mediated pyroptosis, concurrently using aerobic exercise as an intervention in diabetic *db*/*db* mice. We analyzed the alternations of diabetic gastrocnemius muscle (GAS) and found that MCC950 has the potential to attenuate diabetic muscle atrophy by inhibiting NLRP3-mediated pyroptosis. In addition, the combined MCC950 treatment with exercise exhibited a further significant improvement in muscle atrophy. Notably, this combined treatment reduced the levels of apoptosis and pyroptosis of GAS in comparison to an intervention involving either NLRP3 inhibition alone or only exercise.

T2DM and muscle atrophy are age-related diseases, so the young mice used in the current study do not appear to fully reflect the characteristics of diabetic muscle atrophy. Considering that *db*/*db* mice might become too weak with aging to complete the 12-week exercise intervention, we used relatively young *db*/*db* mice and the atrophic state in the mice was thoroughly identified. Nevertheless, it is also important to explore the effects and mechanisms of MCC950 and exercise on alleviating muscular atrophy in older mice. Our study comprehensively explored the therapeutic effects of MCC950 on pyroptosis and diabetic muscle atrophy including muscle mass, strength and exercise performance, as well as the synergistic effects of MCC950 and exercise intervention. The current study innovatively explored the therapeutic potential and the underlying cellular mechanisms in MCC950 for the treatment of diabetic muscle atrophy and for the first time investigated the synergistic effects of MCC950 and aerobic exercise on circulatory inflammatory markers, NLRP3-mediated pyroptosis and diabetic muscle atrophy. Since exercise capacity is reduced when diabetic muscle atrophy occurs, it may not be possible for patients to perform heavy-load resistance exercise. Thus, combining MCC950 with aerobic exercise might be a novel strategy for treatment of the disease.

## 2. Results and Discussion

### 2.1. MCC950 Treatment and Aerobic Exercise Improves Diabetes Glucose Metabolism Levels and Muscle Function

**Figure 1:**
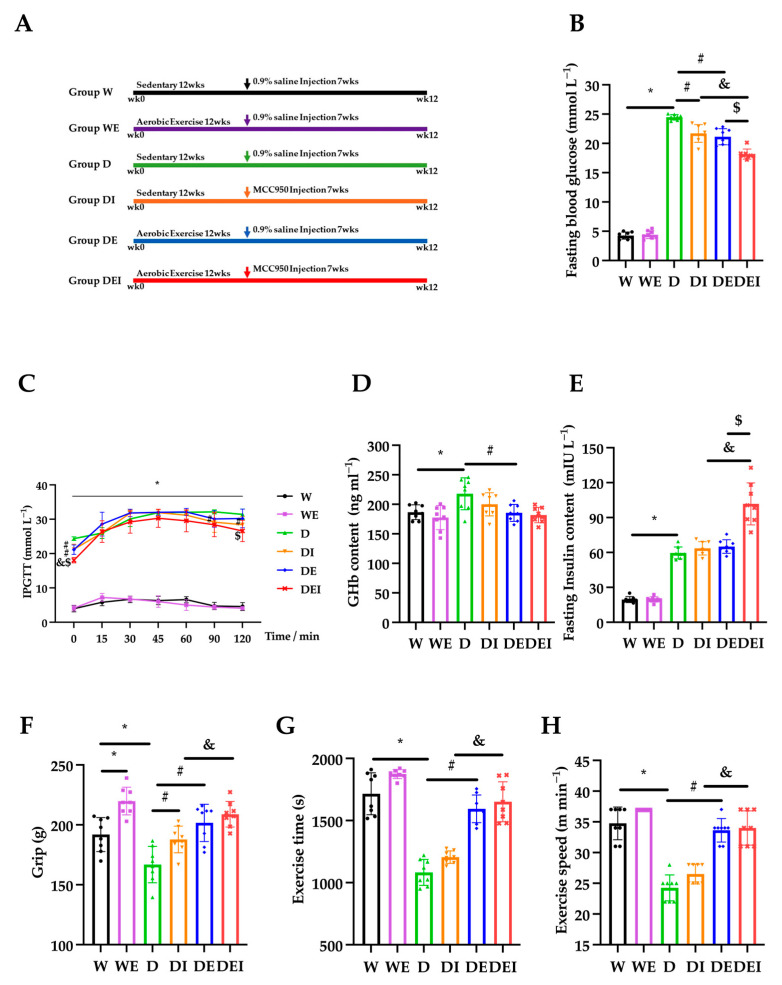
The alternations of glucose metabolism and muscle performance in mice (*n* = 8). The measurements carried on (**A**) intervention protocol; (**B**) fasting blood glucose; (**C**) intraperitoneal glucose tolerance tests (IPGTT); (**D**) glycosylated hemoglobin (GHb); (**E**) fasting insulin content; (**F**) grip strength; (**G**) exercise time and (**H**) exercise speed after the intervention in the wide-type control group (W), wild-type aerobic exercise group (WE), *db*/*db* control group (D), inhibitor MCC950 treatment *db*/*db* group (DI), aerobic exercise *db*/*db* group (DE) and MCC950 treatment plus aerobic exercise *db*/*db* group (DEI). (*) Significant difference compared to the W group; (#) significant difference compared to the D group; (&) significant difference compared to the DI group; ($) significant difference compared to the DE group (*p* < 0.05).

The mice were randomly grouped into six groups: (1) wild-type control group (W); (2) wild-type aerobic exercise group (WE); (3) *db*/*db* control group (D); (4) inhibitor MCC950 treatment *db*/*db* group (DI); (5) aerobic exercise *db*/*db* group (DE) and (6) MCC950 treatment plus aerobic exercise *db*/*db* group (DEI) (Figure 1A). The data on body weight and food intake during the intervention were collected once per week. In the *db*/*db* control mice, the body weight and food intake were significantly higher than those of the wild groups (*p* < 0.05). The body weight of each diabetes intervention group tended to decrease compared with group D, but there was no significant difference in the later stage of intervention. There was no significant difference in the food intake of each diabetes intervention group compared with group D during the experiment (Figure S1A,B).

To assess the capacity of blood glucose regulation in the mice, we measured the fasting blood glucose (FBG), intraperitoneal glucose tolerance tests (IPGTT), glycosylated hemoglobin (GHb) and the fasting insulin content in the mice. The FBG in the *db*/*db* mice (D) was significantly higher than that in the wild-type control mice (W) (*p* < 0.05). Both MCC950 treatment alone (DI) and aerobic exercise only (DE) in the *db*/*db* mice significantly lowered the FBG compared to the *db*/*db* control mice (D). Remarkably, a combination of MCC950 treatment and aerobic exercise (DEI) exhibited a further significant reduction (*p* < 0.05) (Figure 1B). Similarly, IPGTT showed that the diabetic groups exhibited significantly higher blood glucose levels than those in the wild groups (*p* < 0.05). The levels of blood glucose in the DI group at 90 and 120 min were lower than those in the D group, and blood glucose levels in the DEI group at 120 min were lower compared with the DE group (*p* < 0.05) (Figure 1C). The concentrations of GHb and insulin in the D group were significantly higher than those in the W group (*p* < 0.05). The GHb level in the DE group was lower compared with the D group. The concentration of insulin in the DEI group was higher than that in the DI and DE groups (*p* < 0.05) (Figure 1D,E). These data clearly showed that the combination of MCC950 treatment and aerobic exercise has an addictive effect on the improvement of glucose uptake capacity in diabetic mice, indicating a mitigating status in the peripheral tissues such as muscles.

In the C57BLKS/J *db*/*db* mouse used in the current study, hyperinsulinemia is observed at 10 days and blood glucose levels are slightly elevated at 4 weeks after birth. Then, the *db*/*db* mouse develops overt obesity and type-2 diabetes mellitus at 6 weeks, and most of the *db*/*db* mice have a life expectancy of only 10 months [22,23]. Previous studies have indicated that the diabetic *db*/*db* mouse exhibits a similar pathophysiology to and essential characteristics of T2DM, namely obesity, insulin resistance, compensatory hyperinsulinemia and hyperglycemia [24,25], and is commonly used in diabetic muscle atrophy studies [26,27,28]. In addition, the intervention of muscle atrophy in *db*/*db* mice beginning at 6 weeks of age in the current study is similar to previous studies [28,29]. The C57BL/6J *db*/*db* mouse is another mouse model for T2DM. Nevertheless, in our preliminary experiment, this model had a mild increase in blood glucose at the age of 6 weeks, and then the blood glucose started to decline after 10 weeks of age. In the current study, only male *db*/*db* mice were used in order to reduce heterogeneity caused by gender difference.

Next, we carried out the grip strength and exercise performance tests to evaluate muscle functions. The grip strength was significantly increased in the WE group com-pared with that in the W group, and the D group displayed decreased grip strength compared with the W group (*p* < 0.05). The DI and DE groups showed significantly higher strength than that of the D group, and the DEI group exhibited an increased grip strength compared with the DI group (*p* < 0.05) (Figure 1F). The exercise time and speed of the D group were significantly lower than those of the W group and DE group, and the DEI group displayed better exercise performance compared with the DI group (*p* < 0.05) (Figure 1G,H). These results showed that the combined MCC950 treatment with exercise further improved muscle performance.

The current study substantiated that aerobic exercise improves blood glucose regulation and enhances diabetic muscle function (both strength and exercise performance). Although the exact causes of T2DM are not fully understood, many studies have linked impairments in key glucoregulatory functions to the development of this condition [30]. Aerobic exercise can effectively reduce fasting blood glucose; one of the possible reasons is that aerobic exercise has been reported to increase GLUT4 protein levels in skeletal muscle, suggesting that it could improve muscle contraction-stimulated muscle glucose transport [30]. One study has also reported that aerobic exercise improved mitochondrial enzymes, alleviated muscle atrophy and increased oxidative stress [31]. These beneficial effects of aerobic exercise may be due to the activation of AMPK [32].

MCC950 is a potent, specific inhibitor of NLRP3, the mechanism of which is the inhibition of ATPase activity in the NLRP3 inflammasome. MCC950 displays multiple pharmacological properties [33]. Studies have found that MCC950 promotes GLUT4 translocation in skeletal muscle, reduces NLRP3 inflammasome activation, improves insulin resistance in obesity [34], improves muscle function and prevents inflammation [35]. MCC950 treatment for 12 weeks improvead insulin sensitivity in a mouse model of frontotemporal dementia [36]. In the current study, MCC950 treatment improved glucose uptake capacity and muscle strength.

### 2.2. MCC950 Treatment and Aerobic Exercise Alleviates the Degree of Muscle Atrophy

**Figure 2:**
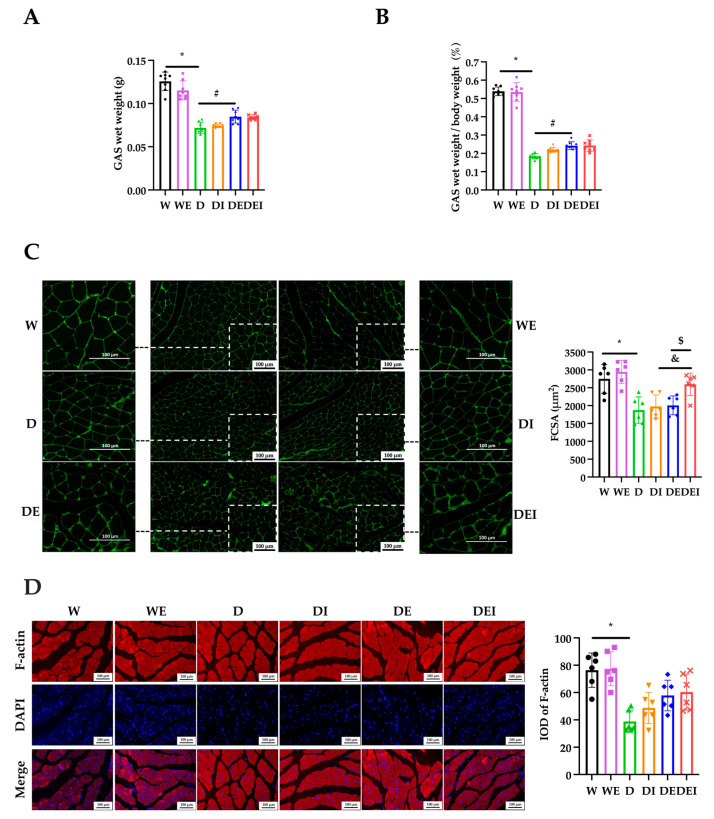
The degree of muscle atrophy in GAS after the intervention. (**A**) The GAS wet weight and (**B**) the ratio of GAS wet weight to body weight (*n* = 8); (**C**) IF staining of Laminin (×200) (left) and statistics of fiber cross-sectional area (FCSA) (*n* = 6); (**D**) IF staining (×200) (left) and the integrated option density (IOD) of F-actin (*n* = 6). (*) Significant difference compared with the W group; (#) significant difference compared with the D group; (&) significant difference compared with the DI group; ($) significant difference compared to the DE group (*p* < 0.05).

To estimate the effects on muscle atrophy in these treated mice, we measured the GAS wet weight, the ratio of the GAS wet weight to body weight and carried out immunostaining of Laminin and F-actin. The GAS wet weight in the D group was significantly lower than in the W group, and that in the DE group was higher in comparison with the D group (*p* < 0.05) (Figure 2A). The ratio of GAS wet weight to body weight in the D group was significantly lower than that in the W group, and that in the DE group was significantly higher than that in the D group (*p* < 0.05) (Figure 2B). The fiber cross-sectional area (FCSA)—indicated by immunofluorescent stains of Laminin—in the D group was significantly lower than the W group, indicating that muscle atrophy had developed in the *db*/*db* mice. Interestingly, a significantly increased FCSA was observed in the DEI group in comparison with the DI group or DE group (*p* < 0.05) (Figure 2C). The integrated option density (IOD) values of F-actin in the D group were lower than those in the W group (*p* < 0.05) but tended to recover after the combination treatment (Figure 2D).

Laminin is a major constituent of the basement membrane. The FCSA indicated by the immunofluorescent staining of Laminin is commonly used to evaluate skeletal muscle atrophy [37]. Studies showed that inflammation induced by lipopolysaccharide (LPS) resulted in a lower cell diameter, and MCC950 alleviated the LPS-triggered myotube growth impairments [17]. In Dapagliflozin-induced muscle atrophy in diabetic rats, after six weeks of aerobic exercise intervention, the FCSA showed a trend toward improvement, although there was no significant difference [38]. Similarly, in the current study, the FCSA demonstrated a tendency to increase in the groups with MCC950 treatment alone or exercise only, although without a significant increase. However, the combined treatment showed an evidently increased FCSA compared with only MCC950 intervention or exercise.

F-actin plays a vital role in cytoskeletal rearrangement and contractile function [39]. Immunofluorescent imaging showed that in the diabetic groups, the expression of F-actin was significantly decreased, and the distribution and regular arrangement of F-actin was disrupted, indicating an attenuated muscle contractile function. The MCC950 treatment or exercise groups showed a trend toward the amelioration of the expression of F-actin, though without a significant difference.

### 2.3. MCC950 Treatment and Aerobic Exercise Moderates the Levels of Apoptosis and Pyroptosis

**Figure 3:**
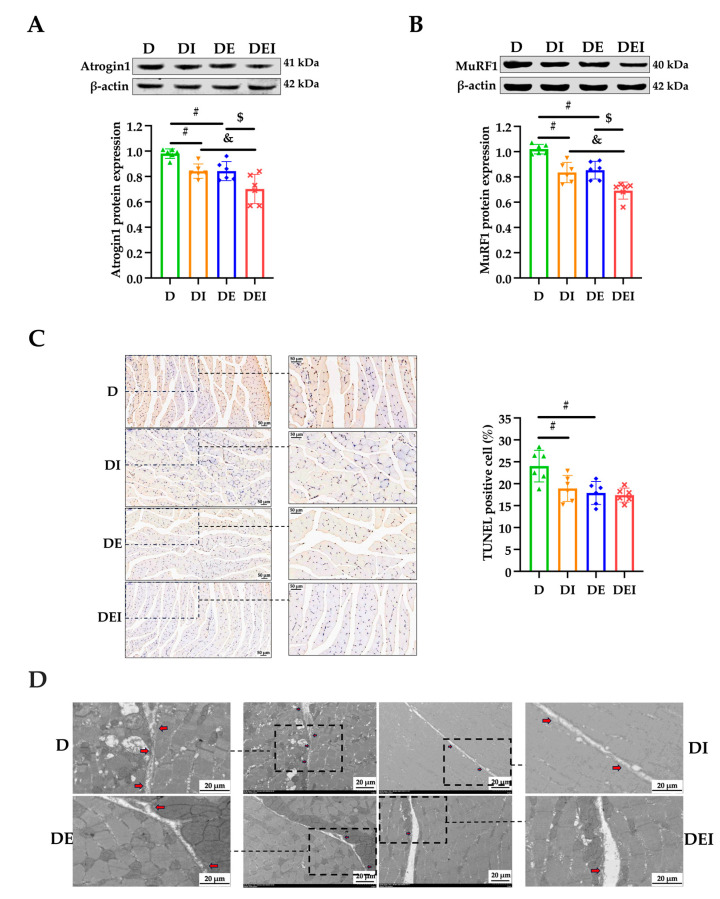
The levels of apoptosis and pyroptosis in GAS in *db*/*db* mice after the intervention (*n* = 6). (**A**) The protein expressions of Atrogin1; (**B**) the protein expressions of MuRF1 and (**C**) the TUNEL staining (×200) (left) and the rate of apoptosis in GAS. (**D**) The presentative transmission electron micrographs (TEM) images show the cell punching in the different groups. The red arrow indicates the cell punching area in the zoomed pictures; (#) significant difference compared with the D group; (&) significant difference compared with the DI group; ($) significant difference compared with the DE group (*p* < 0.05).

To evaluate protein catabolism, we detected expression levels of MuRF1 and Atrogin1, which are muscle-specific E3 ubiquitin ligases, and are vital regulators of the ubiquitin-proteasome pathways (UPP)-mediated protein degradation in skeletal muscle. The protein expression levels of Atrogin1 and MuRF1 in the DI and DE groups were significantly lower than those in the D group, and the expression in the DEI group was significantly decreased compared with the DI and DE groups (*p* < 0.05) (Figure 3A,B), showing that MCC950 treatment or aerobic exercise alleviated diabetic muscle proteolysis, and that the combination of these two interventions manifested better effects for preventing protein degradation.

Muscle atrophy caused by T2DM has been widely documented, but the pharmacological targets for its treatment have not been clarified. It is recognized that the activation of proteolytic ubiquitin-proteasome pathways (UPP) induced by chronic inflammation is involved in the pathogenesis of muscle atrophy [40]. NF-κB, an inflammatory transcription factor, can translocate into the nucleus under certain pathological conditions and upregulate the transcriptional activity of MuRF1 [6]. In non-affected individuals, UPP are activated after acute exercise to clear damaged proteins, while at the same time, the protein synthesis pathway is activated, to prevent muscle atrophy. In inflammatory diseases such as T2DM, long-term exercise training downregulates UPP by inhibiting inflammation, which may be the key to preventing muscle atrophy [41]. In our study, muscle strength, exercise performance, GAS wet weight, wet weight percentage, FCSA as well as the protein expression of F-actin in diabetic mice were decreased and reversed after exercise training (Figure 1 and Figure 2). At the same time, exercise decreased the protein expression of Atrogin1 and MuRF1 (Figure 3A,B).

Recent studies have found that NLRP3 is an important regulator of skeletal muscle metabolism, and a growing amount of evidence shows that the NLRP3 inflammasome is involved in the pathogenesis and development of inflammation-related skeletal muscle atrophy [11,42]. Although there is little evidence showing the relationship between exercise and NLRP3 inflammasome activity in skeletal muscle, accumulating data suggests that exercise training improves cardiac function by suppressing inflammasome activity and cell death in the myocardium [43,44]. Our study found that exercise, and/or inhibition of NLRP3, increased muscle strength and exercise performance, reduced the protein expression levels of Atrogin1 and MuRF1 (Figure 3A,B) and further found that the combined effect of the two methods was best, indicating that NLRP3 may be a key factor in exercise for relieving diabetic muscle atrophy (Figure 2C). Although the role of NLRP3 in the exercise regulation of diabetic skeletal muscle is still unclear, recent studies have confirmed that the NLRP3 inflammasome is related to the pathogenesis of T2DM, and that by inhibiting inflammasome activation in adipose tissue and the liver, it can enhance insulin signaling [45]. Exercise training may reduce insulin resistance and liver injury in elderly prediabetic subjects by inhibiting the activity of the NLRP3 inflammasome [46].

Next, we used TUNEL staining to assess the improvement of apoptotic status with the treatments. The apoptosis levels in the DI and DE groups were significantly decreased compared to the control *db*/*db* mice (*p* < 0.05) (Figure 3C). The apoptosis level tended to continually decrease in the DEI group but did not reach significance. To observe the formation of pores on the cell membrane during pyroptosis and to appraise pyroptosis levels, transmission electron micrographs (TEM) were conducted. From the TEM images, we observed that the number of cells perforated in each intervention group was reduced compared with the D group (Figure 3D). The number appeared to be reduced even more in the DEI group.

### 2.4. MCC950 Treatment and Aerobic Exercise Downregulates the Expression of Pyroptosis-Related Proteins

**Figure 4:**
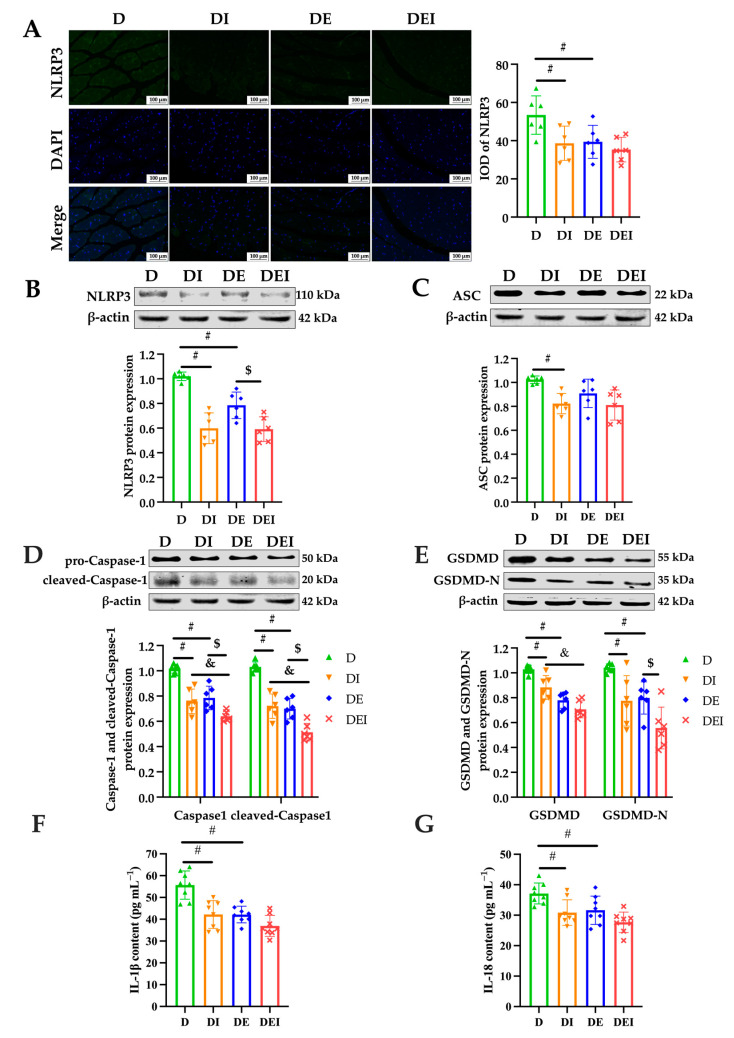
The measurement of pyroptosis-related markers after intervention in the mice. (**A**) IF staining (×200) (left) and the IOD of NLRP3 in GAS of *db*/*db* mice (*n* = 6). The protein expression levels of (**B**) NLRP3; (**C**) ASC; (**D**) Caspase-1 and (**E**) GSDMD were detected by western blot in the GAS of *db*/*db* mice (*n* = 6). Serum inflammatory markers (**F**) IL-1β and (**G**) IL-18 by ELISA (*n* = 8). (#) Significant difference compared with D group; (&) significant difference compared with DI group; ($) significant difference compared with DE group (*p* < 0.05).

To further evaluate which signaling molecules in pyroptosis are activated during the treatment, we proceeded to check the protein expressions of NLRP3, ASC, Caspase-1 and GSDMD and the serum inflammatory markers IL-1β and IL-18. First, the expression of NLRP3 by immunostaining showed that all groups with the treatment were significantly lower than that in the *db*/*db* control group (*p* < 0.05) (Figure 4A). In parallel, the protein expressions of NLRP3 obtained by the western blot appeared as a similar pattern, but the DEI group showed lower levels of NLRP3 compared with the DE group (*p* < 0.05) (Figure 4B). Second, lowered levels of ASC expression in the MCC950-treated group were observed in comparison with the *db*/*db* control group (*p* < 0.05) (Figure 4C). This is the only significant change that was obtained regarding the ASC expression.

Intriguingly, Caspase-1 and cleaved-Caspase-1, the representatives of the activation of pyroptosis, were significantly reduced in the double-treated group (DEI) compared to the inhibitor alone (DI) and exercise only (DE). As expected, the inhibitor-treated group and exercise groups also displayed significantly decreased levels in the expression of Caspase-1 and cleaved-Caspase-1 than those in the *db*/*db* control group (*p* < 0.05) (Figure 4D). Further, the protein expression of GSDMD, which cleaved by active caspases to liberate the N-terminal domain that determines pyroptosis, showed similar results (*p* < 0.05). Compared to the *db*/*db* control group, the inhibitor-treated group exhibited lower levels of GSDMD-N protein expression, and those of the double-treated group were lower in comparison with the exercise group (*p* < 0.05) (Figure 4E).

Finally, to assess the overall inflammatory status, the serum IL-1β and IL-18 were measured in the mice. Compared to the *db*/*db* control group, all groups with the treatment showed lowered serum concentrations of IL-1β and IL-18 (*p* < 0.05) (Figure 4F,G), indicating improved inflammatory conditions. The DEI group tended towards a further decrease compared to the DI and DE groups but failed to reach significance.

Intracellular Toll-like receptors (TLR) regulate muscle fiber size and tissue inflammation and are therefore significant influencers of muscle function [47,48]. TLR activate NLRP3 and its inflammasome (composed of NLRP3, an apoptosis-associated speck-like protein containing a caspase-recruitment domain (ASC) and the cysteine protease Caspase-1 (pro-Caspase-1)) by activating NF-κB to produce active Caspase-1, which mediates the maturation and secretion of IL-1β and IL-18. Moreover, activated Caspase-1 induces Gasdermin D (GSDMD)-mediated pore formation, osmotic swelling and plasma membrane rupture, leading to a series of inflammatory responses, known as pyroptosis. Pyroptosis is manifested by membrane perforation and continuous cellular swelling until the rupture of the cell membrane, resulting in the release of pro-inflammatory intracellular contents, such as IL-1β and IL-18, which lead to local or systemic inflammatory responses [49]. The pyroptotic signaling pathway is mediated by inflammasome assembly, with GSDMD cleaved by upstream Caspase-1 as well as IL-1β and IL-18 release [50]. Thus, the current study examined serum IL-1β and IL-18 content to investigate the circulating inflammation states. To further determine the role of NLRP3 in reducing skeletal muscle inflammation by mediating pyroptosis, we explored the expression of pyroptosis-related proteins. We found that the degree of apoptosis and pyroptosis (Figure 3C,D), and the expression of pyroptosis-related proteins in diabetic skeletal muscle and circulating inflammation, were reduced by exercise and/or inhibition of NLRP3 (Figure 4).

A large body of evidence suggests that inflammation is involved in the pathogenesis of T2DM. The levels of inflammatory cytokines such as CRP, TNF-α, IL-1β and IL-6 have been positively correlated with the incidence of T2DM [51]. Considering that the normalization of blood glucose and lipids is not enough to eliminate the harm of clinical outcomes and complications of T2DM, it is necessary to analyze the long-term effects of exercise on diabetic inflammatory changes. Reducing the level of inflammation by exercise may be more decisive in controlling and alleviating the development of T2DM than improving glucolipid metabolism [52]. Aerobic exercise is more effective than resistance training in reducing the levels of inflammatory factors. In our study, exercise significantly reduced the content of inflammatory factors IL-1β and IL-18 (Figure 4F,G). Studies have also confirmed that exercise relieves muscle atrophy by alleviating muscle inflammation in *db*/*db* mice. It is further believed that long-term regular exercise may reduce the expression of MuRF1 by inhibiting the activation of NF-κB in the muscle [6]. In addition, exercise relieves insulin resistance and decreases blood glucose levels, reduces the accumulation of visceral fat and increases antioxidant capacity to reduce the accumulation of reactive oxygen species (ROS), eventually inhibiting inflammation related to T2DM. These effects contribute to the mechanisms for alleviating muscle atrophy [47,52]. In our study, exercise reduced the NLRP3 signaling pathway in GAS muscle; thus, exercise might moderate pyroptosis by inhibiting the ROS/NF-κB/NLRP3/Caspase-1/GSDMD-mediated pyroptotic pathway. MCC950, as the NLRP3-specific inhibitor, also alleviates pyroptosis by inhibiting the NLRP3/Caspase-1/GSDMD-mediated pyroptotic pathway. The combination of these two interventions may jointly activate the pyroptosis pathway and demonstrate better therapeutic potential than MCC950 treatment alone or exercise only.

Although the mechanism in aerobic exercise which alleviates apoptosis in diabetic muscle atrophy is still unclear, evidence has demonstrated that insulin and insulin-like growth factor-1 (IGF-1) signaling pathways play an important role in the phosphorylation of phosphoinositide 3-kinase (PI3K)/protein kinase B (Akt)-mediated apoptosis and protein synthesis [53,54]. Insulin and IGF-1 secretion are reduced in T2DM, and this suppression of IGF-1 pathways results in decreased protein synthesis and increased apoptosis. Studies have reported that exercise upregulates IGF-1 and can attenuate cell apoptosis in myocardial infarction (MI)-induced muscle atrophy by the IGF-1R-PI3K/Akt pathway [55]. In addition, aerobic exercise also improves metabolic states via mitophagy and apoptosis in *db*/*db* mice [56]. NLRP3 activation has been reported to be closely associated with cell apoptosis [57]. NLRP3 conditional knockout (cKO) mice attenuate apoptosis in denervated muscle atrophy [58]. Inhibiting NLRP3 with MCC950 alleviates apoptosis in the retinal endothelial cells in diabetic retinopathy [59]. Another study reported that pretreatment with MCC950 upregulated levels of HIF1A, BECN1, BNIP3 and LC3B-II in iohexol-treated HK-2 cells and indicated that inhibiting NLRP3 alleviates apoptosis via the activation of HIF1A- and BNIP3-mediated mitophagy in contrast-induced acute kidney injury [60]. Therefore, MCC950 treatment might synergize with exercise to attenuate apoptosis via the multiple pathways mentioned above in the diabetic atrophy in mice.

In the current study, we used MCC950 to treat pyroptosis and diabetic muscle atrophy in mice. We found that the MCC950 treatment improved muscle performance, downregulated proteolytic proteins, significantly reduced NLRP3-mediated inflammatory factors such as ASC, pro-Caspase-1, cleaved-Caspase-1, GSDMD and GSDMD-N, downregulated the serum content of IL-1 β and IL-18 and alleviated pyroptosis in atrophic GAS. In summary, MCC950 reduced NLRP3-mediated pyroptosis over-activation, and it reversed muscle homeostasis and muscle function in *db*/*db* mice. Correspondingly, these findings provide further evidence for the role of NLRP3-mediated pyroptosis in the pathogenesis of diabetic muscle atrophy and show that MCC950 can moderate these effects. MCC950 is thus a novel potential therapeutic for the prevention and treatment of diabetic atrophy. Moreover, these results demonstrate a remarkable benefit of the inhibition of pyroptosis towards the anti-inflammatory results of aerobic exercise in the diabetic *db*/*db* mice.

**Figure 5:**
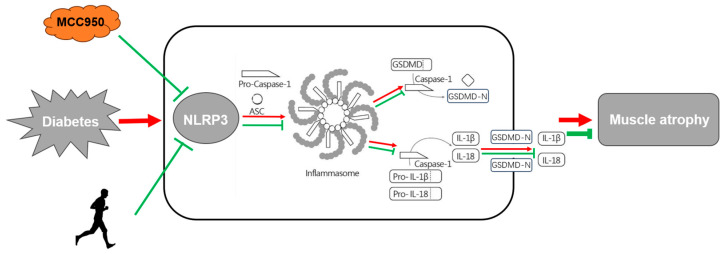
Depiction of anti-inflammatory pathways by NLRP3-related pyroptosis and aerobic exercise in muscle atrophy in diabetic *db*/*db* mice. Note that the red arrows indicate the pro-atrophic effects under diabetic conditions whilst the green arrows represent anti-atrophic effects resulting from NLRP3 inhibitor MCC950, aerobic exercise or both.

In this study, we corroborate that *db*/*db* mice exhibit a typical phenotype of muscle atrophy, such as impaired grip strength, decreased muscular endurance, decreased muscle structural marker proteins and increased proteolytic proteins. Notably, we found that MCC950 treatment significantly reduced the atrophic parameters that had deteriorated in the *db*/*db* mice. Moreover, MCC950 administration inhibited NLRP3, ASC, Caspase-1, GSDMD and prevented pyroptosis in atrophic GAS. Mechanistically, the above changes induced by MCC950 were attributed to inhibiting the NLRP3-mediated pyroptosis. In addition, we found an enhanced effect on glucose uptake capacity and muscle performance exhibited in the group which combined MCC950 treatment with exercise. This combined treatment significantly increased the FCSA, compared to the group with inhibitor treatment alone or exercise only. Indeed, the synergetic effect of MCC950 treatment on exercise elicits a significant reduction of NLRP3-mediated inflammatory factors such as cleaved-Caspase-1 and GSDMD-N in atrophic muscles (as shown in Figure 5). These data demonstrate a novel strategy for the treatment of diabetic muscle atrophy by inhibiting NLRP3-mediated pyroptosis with MCC950 and aerobic exercise.

## 3. Materials and Methods

### 3.1. Animals

Forty male C57BLKS/JGpt *db*/*db* mice (6-week-old, weighing 36.53 ± 2.27 g) and sixteen male C57BLKS/JGpt m/m mice (6-week-old, weighing 18.73 ± 0.73 g) were purchased from GemPharmatechTM Co., Ltd. (Jiangsu, China) All mice were housed under standardized conditions with a consistent temperature of 22 ± 2 °C, relative humidity of 50–70% and a 12 h light/dark cycle; in addition, all mice had ad libitum access to water and standard rodent chow (purchased from Beijing HFK Bioscience Co., Ltd., Beijing, China) and were 3–4 mice per cage (294 × 190 × 125 mm). All experimental protocols and animal handling procedures were approved by the Ethics Committee of Sports Science Experiment Ethics Committee of Beijing Sport University (Ref. No: 2020147A).

The mice were allowed to acclimate for a week before formal experiments. The body weight and food intake of each mouse were recorded via electronic scales each week during the experiments. Blood samples were taken from the tail vein of mice after fasting for 12 h and tested using a glucometer (Accu-Chek Performa; Roche, Mannheim, Germany) to determine that the mice were diabetic before all experiments involving *db*/*db* mice were performed.

After one week of adaptation, sixteen m/m mice were randomly divided into two groups: wild-type control group (W) and wild-type aerobic exercise group (WE), *n* = 8. Forty *db*/*db* mice were randomly divided into four groups: (1) *db*/*db* control group (D), (2) inhibitor MCC950 treatment *db*/*db* group (DI), (3) aerobic exercise *db*/*db* group (DE) and (4) MCC950 treatment plus aerobic exercise *db*/*db* group (DEI), *n* = 10. For MCC950 administration, both the DI group and the DEI group at 12 weeks old received intraperitoneal injections of MCC950 (MedChem Express, Shanghai, China), at a dose of 10 mg kg^−1^ [61,62,63] body weight three times per week [64,65] (Monday, Wednesday, and Friday at 10–11 am) for seven weeks before the mice were euthanized. Other mice received an injection of 1 mL of 0.9% saline in an identical manner.

### 3.2. Mice Training Protocol

Mice assigned to the exercise groups underwent acclimation with treadmill exercise for 3 days (6 m min^−1^, 20 min d^−1^) at 0% grade. Then, they underwent a 12-week training protocol [66] consisting of continuous treadmill running for 5 d w^−1^, 60 min d^−1^ at 0% grade at a final intensity equivalent to ~50% of the maximal speed-of-exhaustion (MSE) started at 7 weeks old. For acclimatization, treadmill speed and exercise duration were gradually increased over the first 2 weeks. For example, the mice ran at 6 m min^−1^ (*db*/*db* mice) and 9 m min^−1^ (m/m mice) (5 d w^−1^, 30 min day^−1^) for 1 week, and the speed was increased to 8 m min^−1^ (*db*/*db* mice) and 11 m min^−1^ (m/m mice) (5 d w^−1^, 45 min d^−1^) in the following week. After 2 weeks of acclimatization, the exercise training achieved a target of 60 min of exercise per day at a speed of 10 m min^−1^ (*db*/*db* mice) and 13 m min^−1^ (m/m mice). The daily exercise consisted of a 10 min warm-up, a 45 min run, and a 5 min cool-down. The control groups were kept under standard, sedentary conditions for the same duration.

### 3.3. Intraperitoneal Glucose Tolerance Tests (IPGTT)

After 12 weeks of training, the mice were subjected to an IPGTT to investigate the glucose uptake capacity. Briefly, mice were fasted for 6 h and then injected intraperitoneally with 0.5 g kg^−1^ glucose. Vein blood was obtained from the tail tip at 0 (prior to glucose administration), 15, 30, 45, 60, 90 and 120 min after glucose administration for the measurement of glucose levels via a glucometer [67].

### 3.4. Muscle Sample Collection

This sample collection was performed 48 h after the completion of the last running session, to ensure that measurements reflected the long-term effects of the 12-week training program, rather than the immediate effects of the last exercise. After 12 h fasting, mice were weighed by an electronic weighing scale, anesthetized by Urethane, had blood collected and then were quickly euthanized by cervical dislocation. The bilateral gastrocnemius muscle (GAS) tissues were harvested immediately and weighed, and then cut into two pieces for molecular biological and histological analysis. Specifically, the medial head of the GAS was used for molecular biological analysis and the lateral head of the GAS for histological analysis. The GAS is a muscle composed of a mixed fiber-type population (type I, type IIa, type IIb). Deeper regions of both medial and lateral GAS, which are adjacent to the soleus muscles, contain numerous type I and type IIa fibers. Superficial regions generally contain numerous type IIb fibers [68]. The blood samples and muscle samples for biological analysis were separately stored at −80 °C until analyzed.

### 3.5. Serum Glucose Profiles

Blood samples were collected by cardiac puncture from overnight-fasted mice 48 h after the last exercise session. After clotting, the serum was separated by centrifugation 3500 rpm for 15 min at 4 °C. Serum glucose was measured by the automatic biochemical analyzer [69] (Mindray, BS-350E) using the specific assay kits (Mindray Co., Ltd., Shenzhen, China).

### 3.6. Serum Glycosylated Hemoglobin (GHb), Insulin and Inflammatory Markers Content

Mouse serum concentrations of GHb [70], insulin [71] and inflammatory markers (IL-1β, IL-18) [72] were assayed using ELISA kits (Jianglai, Shanghai, China), according to the manufacturer’s instructions.

### 3.7. Exercise Performance Test

After the acclimatization period, the mice performed an “Incremental Load Test” to determine the MSE, using a protocol by Ferreira et al. [73] with mild modification. This exercise performance test allowed us to determine the maximal aerobic capacity, pre-scribe aerobic exercise training sessions and examine the effectiveness of exercise training. In brief, the mice were first familiarized with treadmill running for 2 days. The treadmill was equipped with an electrical stimulation grid at the rear of the tracks, and the mice would receive low-frequency electric shocks if they fell off the track. The mice initiated the test at 10 m min^−1^, increasing 3 m min^−1^ every 3 min at 0% grade until exhaustion. Exhaustion was defined as when the mice were unable to remain on the treadmill despite repeated contact with the electric grid (0.4 mA) and gentle encouragement to run for more than 10 s. Once exhaustion was reached, the power of the shock grid was immediately turned off and the mice were removed from the treadmill and returned to their home cage. The speed achieved and time-to-exhaustion were monitored. Based on the former, workloads corresponding to ~50% peak speed were determined. To assess the effectiveness of the training regime and MCC950 administration, this protocol was repeated at the end of the experimental period.

### 3.8. Grip Strength Test

The limb muscle grip strength of mice was measured by a digital grip strength meter (Beijing Zhongshi Dichuang Technology Development Co., Ltd., Beijing, China), as described previously [74]. Briefly, the mice were first acclimated to the use of the meter for 10 min before the test began. To perform a measurement, a mouse was grasped gently at the base of the tail and allowed to grab the metal pull bars with its four paws. The mouse was positioned perpendicular to the bar and pulled backwards by the tail in a horizontal fashion until the grip was lost. The peak force of each measurement was automatically recorded by the apparatus in grams (g). Each mouse was tested for 3–5 trials with a 30 s break, and the average data were used for statistical analysis. Experimenters were blind to the animal groups.

### 3.9. Immunofluorescence (IF) Staining

The protein expression in GAS was determined by immunofluorescence staining to evaluate the levels of Laminin, the FCSA, filamentous actin (F-actin) and NLRP3. GAS was fixed in 4% buffered paraformaldehyde for 2 days, embedded in paraffin and processed for sectioning. GAS paraffin sections were washed with gradient ethanol, rinsed with distilled water and subjected to antigen retrieval. Sections were then quenched for autofluorescence and blocked with 5% bovine serum albumin. Following blocking, sections were stained using Laminin (Servicebio, GB111414, Wuhan, China), F-actin (Abcam, ab130935, Cambridge, UK) and NLRP3 (Cell Signaling Technology/CST, 15101S, Danvers, MA, USA) as primary antibodies overnight at 4 °C. The next day, sections were incubated with secondary antibodies (Servicebio, GB21301/21303/25303) for 50 min at room temperature. After the slice sections were washed, DAPI was used to counterstain cell nuclei. Finally, the sections were mounted with a coverslip and photographed under a confocal laser scanning microscope (Nikon Eclipse C1, Tokyo, Japan). Images (three different fields/section) acquired were third person blinded and analyzed using IPP 6.0 software to measure the integrated optical density (IOD) value of the selected area, indicating the intensity of immunopositivity [58].

### 3.10. TdT-Mediated dUTP-Biotin Nick End Labeling (TUNEL) Staining

GAS paraffin sections were first deparaffinized and rehydrated, and then retrieved with antigen, permeabilized and inactivated endogenous peroxidase and equilibrated at room temperature. Subsequently, the sections were incubated in TUNEL reaction solution and mixed with reagent Streptavidin-HRP and TBST, DAB developed and counterstained in nuclei. Next, the slices were dehydrated, briefly dried, mounted with a resin mounting medium and then underwent microscopic inspection. Images of three different fields of view were selected from the center and four corners of each section, and the percentage of TUNEL-positive cells was counted using Image J Ver 1.52a software [75].

### 3.11. Transmission Electron Micrographs (TEM)

GAS was isolated from mice, cut into small muscle blocks, and then immediately fixed using 2.5% glutaraldehyde (Solarbio, Beijing, China). Tissue samples were fixed in 1% osmium tetroxide for 2 h and then dehydrated through a graded series of acetone. Following this, the samples were embedded with epoxy resin and polymerized. The blocks were then cut into 70 nm thick sections. After negative staining of uranyl acetate and lead citrate, the images were acquired by a transmission electron microscope (HT7800, Hitachi, Tokyo, Japan) [76].

### 3.12. Western Blot

The total protein of GAS was extracted and quantified using a BCA protein quantification kit (Thermo Fisher Scientific, Waltham, MA, USA). Protein samples were loaded into 15 wells (20 μg total protein per well) of 4–12% Bis-Tris gradient gels (NP0336BOX, Invitrogen, Waltham, MA, USA). After separation by electrophoresis, proteins were transferred to nitro-cellulose Regular Stack (IB23001, Invitrogen). Membranes were blocked with Odyssey blocking buffer (LI-COR), and then incubated with anti-NLRP3 (CST, 15101S), anti-ASC (Novus Biologicals, NBP1-78977, Centennial, CO, USA), anti-Caspase-1 (CST, 2225S), anti-GSDMDC1 (Santa Cruz, sc-393656, Santa Cruz, CA, USA), anti-MuRF1 (Santa Cruz, sc-398608) and anti-Atrogin1 (Abcam, ab168372) antibodies overnight at 4 °C. Then, after being washed four times (8 min each) by Tris-buffered saline (TBS) containing Tween-20 (TBST), the membranes were incubated with goat anti-rabbit or anti-mouse IgG secondary antibodies (LI-COR, 926-68071/926-32210, Lincoln, NE, USA) at 25 °C for 1 h. Next, the membranes were washed with TBST four times (8 min each) and then TBS twice (5 min each). The protein bands were detected by a near-infrared spectroscopy detection system (Odyssey CLX, LI-COR). All bands were analyzed semi-quantitatively using Image Studio Ver 5.2 software [58].

### 3.13. Statistical Analyses

Statistical analysis was performed using SPSS 22.0. All data are presented as mean ± standard error of the mean. The student’s *t*-test or Two-Way ANOVA was used to determine the significance value. The Bonferroni method was used as a post hoc test. The significance level was set at *p* < 0.05.

## 4. Conclusions

In this study, whilst a diabetic muscle atrophy phenotype in the *db*/*db* mice was thoroughly examined, related effectors such as aging might have been overlooked. The *db*/*db* mice were used at six weeks of age. Though the mice had already started to develop diabetic status, T2DM is an age-related disease, and as such, these mice were still relatively young. Therefore, in the future, further exploration of the effects of and mechanisms in aerobic exercise alleviating muscular atrophy could be examined in older mice or high-fat diet-induced models. In addition, in vivo data such as intramuscular TG analysis, as well as body composition assessment through Magnetic Resonance Imaging (MRI) or muscle mass through Dual-emission X-ray Absorptiometry (DXA) will provide more in vivo dynamic information regarding muscle atrophy in diabetic mice.

MCC950, a specific, small-molecule NLRP3 inhibitor, has the potential to alleviate diabetic muscle atrophy by inhibiting NLRP3-mediated pyroptosis. A combined MCC950 treatment with exercise exhibits a further improvement in glucose uptake capacity, muscle performance and eventually muscle atrophy. Moreover, this combined treatment also reduces NLRP3-mediated pyroptosis in the atrophic gastrocnemius muscle.

## Data Availability

The datasets used and/or analyzed during the current study are available from the corresponding author on reasonable request. The data are not publicly available due to that the authors would like the research data remains private.

## Supplementary Materials

The following supporting information can be downloaded at: https://www.mdpi.com/article/10.3390/molecules29030712/s1, Figure S1: The body weight, food intake during intervention in mice (*n* = 8).
