# Supplementary material for: MCC950 Ameliorates Diabetic Muscle Atrophy in Mice by Inhibition of Pyroptosis and Its Synergistic Effect with Aerobic Exercise
Source: Molecules. 2024 Feb 4;29(3):712. doi: 10.3390/molecules29030712 (PMC10856337; doi:10.3390/molecules29030712)
Supplement: Supplementary file 1 [file molecules-29-00712-s001.zip › molecules-2813002-supplementary.pdf]

Supplementary Materials

MCC950 Ameliorates Diabetic Muscle Atrophy in Mice by Inhibition of Pyroptosis and Its Synergistic Effect with Aerobic Exercise

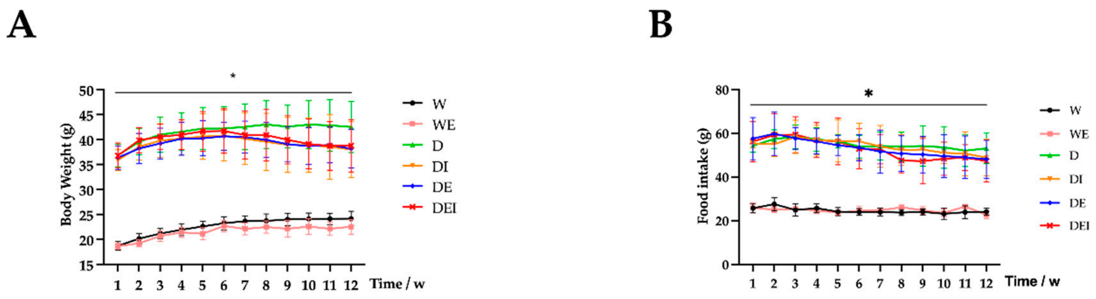

Figure S1. The body weight, food intake during intervention in mice (n = 8). (**A**) the body weight and (**B**) the food intake during the intervention. * Significant difference compared with W group ($p < 0.05$).
